# Bilirubin Induced Encephalopathy

**Published:** 2020

**Authors:** Parvaneh KARIMZADEH, Minoo FALLAHI, Mohammad KAZEMIAN, Naeeme TASLIMI TALEGHANI, Shamsollah NOURIPOUR, Mitra RADFAR

**Affiliations:** 1 Pediatric Neurology Research Center, Research Institute for Children’s Health, Shahid Beheshti University of Medical Sciences, Tehran, Iran; 2 Pediatric Neurology Department, Mofid Children’s Hospital, Faculty of Medicine, Shahid Beheshti University of Medical Sciences, Tehran, Iran; 3 Neonatal Health Research Center Research Institute for Children’s Health, Shahid Beheshti University of Medical Sciences, Tehran, Iran; 4 Shahid Beheshti University of Medical Sciences, Mahdieh Hospital, Tehran, Iran; 5 Shahid Beheshti University of Medical Sciences, Imam Hossein Hospital, Tehran, Iran

**Keywords:** Bilirubin Induced Encephalopathy, Kernicterus, Neonatal Jaundice

## Abstract

Hyperbilirubinemia is one of the most common neonatal disorders. Delayed diagnosis and treatment of the pathologic and progressive indirect hyperbilirubinemia lead to neurological deficits, defined as bilirubin induced encephalopathy (BIE) (2). The incidence of this disorder in underdeveloped countries is much more than developed areas. All neonates with the risk factors for increased the blood level of indirect bilirubin are at risk for BIE, especially preterm neonates which are prone to low bilirubin kernicterus . BIE can be transient and acute (with early, intermediate and advanced phases)or be permanent, chronic and lifelong ( with tetrad of symptoms including visual (upward gaze palsy), auditory (sensory neural hearing loss), dental dysplasia abnormalities, and extrapyramidal disturbances (choreoathetosis cerebral palsy).Beside the abnormal neurologic manifestations of the jaundiced neonates ,brain MRI is the best imaging modality for the confirmation of the diagnosis. Although early treatment of extreme hyperbilirubinemia by phototherapy and exchange transfusion can prevent the BIE, unfortunately the chronic bilirubin encephalopathy does not have definitive treatment.

## Introduction

Hyperbilirubinemia is one of the most common neonatal disorders at the first days and weeks of life (1) Delayed diagnosis and treatment of the pathologic and progressive indirect hyperbilirubinemia can cause permanent neurological deficits, defined as bilirubin induced encephalopathy (BIE) (2). The main problems in this disorder include: central nervous system insult, auditory, visual, dental, neuromotor, and language impairments (3). Depends on the duration of severe hyperbilirubinemia in the neonate and initiation time of treatment modalities (phototherapy and exchange transfusion), the adverse effects of hyperbilirubinemia and brain cellular damage can be transient and minimal or permanent and severe. The symptoms of this disorders can be divided into acute or chronic phases (4). 

 Although the incidence of jaundice is high in the neonatal population, the rate of severe hyperbilirubinemia leading to chronic bilirubin encephalopathy is low and kernicterus is relatively an uncommon disorder in the developed countries, however, in underdeveloped areas of the world, its occurrences are relatively more common **(**5**). **High values of indirect free bilirubin in the blood which couldn’t bound to albumin can transfer from the blood-brain barrier and precipitate in the brain cells and disturb the normal functions of central nervous systems** (**6**).** Regarding of the Rh incompatibility between the mother and neonate as the most common cause of severe hyperbilirubinemia, and the introduction of RhoGam(anti Rh antibody)since the beginning of 1960, maternal sensitivity to fetal antigens, during pregnancy and after delivery is declined and recently ABO incompatibility is the most important cause of neonatal jaundice** (**7**). **

Given that the severity of hyperbilirubinemia in ABO incompatibility is less than Rh incompatibility, the observation of severe and extreme hyperbilirubinemia leading to BIE is not expected these days although in spite of modern facilities for treatment of neonatal hyperbilirubinemia, continuing the case reports of BIE is concerning. This event is an alarm to the healthcare systems for planning the screening program of hyperbilirubinemia during the golden first hours of life. The most important things is timely diagnosis and the early detection of the mild and invisible hyperbilirubinemia by the healthcare services or parents in order to early intervention and treatment of hyperbilirubinemia. American academy of pediatrics (AAP) recommended the pre-discharge screening of hyperbilirubinemia in all well babies at the nurseries. It is suggested that transcutaneous bilirubin meter as a noninvasive tool used be for this screening** (**8**). **With this screening method, post-discharge visiting of the neonates and early detection of jaundice is scheduled. This review article updates the information about the BIE and had a brief review of the new guidelines for the prevention of this devastating neonatal disorder.

**Definition**

The adverse effect is (neuropathology and clinical finding) of free plasma indirect bilirubin on the nervous system defined as bilirubin induced encephalopathy (BIE). This complication can be transient and reversible or be permanent and lifelong** (**9**).**

Acute manifestations of bilirubin neurotoxicity in early stages in the neonatal periods is, defined as acute bilirubin encephalopathy (ABE) and permanent and chronic sequela of bilirubin toxicity is known as kernicterus.

Not all cases of acute bilirubin encephalopathy progress to kernicterus and not all patients with chronic bilirubin encephalopathy have a previous history of obvious bilirubin encephalopathy during the first days of life. 

**Incidence**

Despite the understanding of the basic pathophysiology of bilirubin toxicity and available treatment modalities of this disorder, unfortunately, bilirubin encephalopathy is reported all over the world, however, the new cases of the disease in underdeveloped countries is much more than the developed ones. We did not have the definite incidence of disease in our country Iran, but the incidence range of kernicterus in developed countries is reported 1 in 40000 live birth (10). In a recent report of Sweden, while the incidence of extreme hyperbilirubinemia needs to exchange transfusion is 50 in 100000 live births, fortunately, the incidence of kernicterus is reported 1.3 in 100000 live births (11). Regarding of the occurrence of low bilirubin kernicterus in preterm infants and overlap of the symptoms of bilirubin toxicity with the other neurological sequelae of prematurity, the exact incidence of BIE in premature neonates is not specified. In a recent study in japan in preterm neonates with gestational age lower than 30 weeks, the incidence of kernicterus reported 1.8 in 1000 live birth** (**12**).**

**Pathophysiology**

Deposition of indirect and unconjugated bilirubin in the brain cells is the main underlying pathophysiologic process of BIE (13).

**Figure-1:**
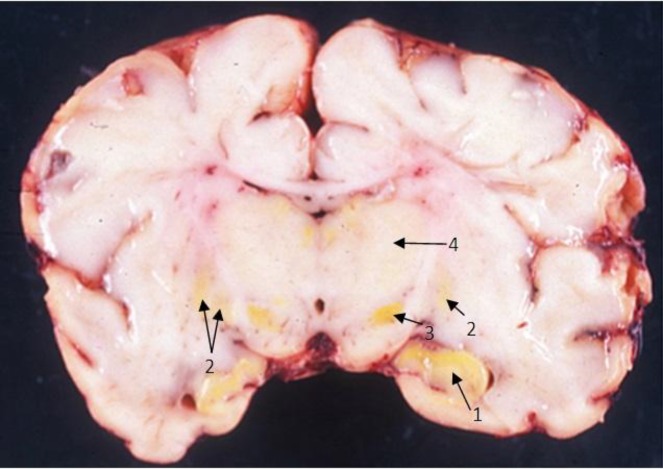
ABE. Coronal section through posterolateral lobes.(From:Zangen S, et al,fatal kernicterus in a girl deficient in G6OD,a paradigm of synergistic heterozygosity.J Pediatri.2009;154:616-619) 1 Hippocampus,2- Basal ganglia ,3-Substantia Nigra ,4-Thalamus

Globus pallidus, basal ganglia, substantia nigra, hippocampus, thalamic nuclei, putamen nuclei, dentate, inferior olives, and cerebellum are the most vulnerable areas of the brain to the toxicity induced by bilirubin with a symmetric pattern of involvement in the mentioned areas. The cranial nerves, primarily the third, fourth, and sixth ones also involved in this disorder (14). Cochlear nuclei, oculomotor and vestibular system involve too. Associated destructive lesions in the white matter and periventricular infarcts have been reported. The cerebral cortex is spared (15) **Intense **yellow coloring of the mentioned areas of the brain in autopsy revealed deposition of indirect bilirubin in these sites. **(**Figure-1**)** In the evaluation of cellular pathology, impairment of the glucose transport system, DNA, protein and neurotransmitter synthesis and activity of many enzymes, iron transport and apoptosis are detected. Degeneration of mitochondria, and membrane alterations of brain cells, cause the irreversible and permanent changes leading to chronic bilirubin encephalopathy** (**16**).**

**Risk factors for bilirubin   induced encephalopathy:**

Although in all neonatal populations, delay diagnosis and treatment of severe hyperbilirubinemia is the main cause of bilirubin encephalopathy, there are many risk factors for the pathologic raising the blood level of indirect bilirubin in CNS. Considering that this disorder, has a multifactorial etiologist, there are other risk factors than bilirubin level, increase the probability of this disease. All underlying disorders for increasing the blood levels of bilirubin such as hemolytic disease of newborns, sepsis, neonatal nonimmune hemolytic disorders such as congenital spherocytosis, G6PD deficiency, congenital and genetic-based disorders for bilirubin metabolism such as Crigler-Najjar and Lucey-Driscoll syndromes, hypoalbuminemia, acidosis, hypoglycemia are known predisposing factors for bilirubin toxicity **(**1**, **17**).**

Regard to the immaturity of the blood-brain barrier (BBB) in the neonatal period and necessitate of the passage of indirect bilirubin from the BBB, for deposition of bilirubin in the brain cell, all disorders with the damage of the BBB or increase its permeability to bilirubin, can be a major risk factor for bilirubin toxicity. Hypoxic-ischemic encephalopathy, prematurity, sepsis, and meningitis are the main underlying disorders with effect by this mechanism. (18) Other risk factors consisted of low birth weight, cephalhematoma or easy bruising, exclusive or unsuccessful breastfeeding, early discharge from the nursery (lower than 24-48 hours of life), lack of predischarge screening of hyperbilirubinemia of neonates (19) .

New research focus on the Genetic tendency for BIE**.** Determination of the underlying genetic tendency, help the healthcare services to select the patient susceptible to BIE for more aggressive management of neonatal jaundice compared to other patients without this genetic tendency (20**).**

**Low bilirubin Kernicterus**

The occurrence of BIE by the bilirubin level lower than the expected plasma level need to exchange transfusion or high intensive phototherapy is, defined as the Low bilirubin Kernicterus. Regard to the multifactorial bases of bilirubin encephalopathy, in this situation, other factors than the sole bilirubin level are the predisposing factors for this disorder (21, 3). Premature neonates are the most vulnerable patients in this condition. Hypoalbuminemia, sepsis, meningitis, hypoxic-ischemic encephalopathy, intraventricular hemorrhage(IVH), periventricular leukomalacia(PVL), the administration of drug competing with albumin binding bilirubin such as ibuprofen (for treatment of patent ductus arteriosus(PDA), or ceftriaxone, are the most important risk factors for low bilirubin kernicterus **(**22).

This condition emphasizes that, not all patients with defined kernicterus had the history of neonatal high bilirubin levels needed to exchange transfusion or phototherapy in the first days of life especially in premature babies. Developmental differences in CNS permeability to bilirubin is the main pathophysiological basis of this defect. **I**n a recent report of kernicterus in the preterm infant, the rate of this complication is reported 2 in 1000 of premature babies with the gestational age lower than 30 weeks, while the incidence of disease in term neonates is 1in 40000 live birth (23). 

There is some research indicated that in term neonates with moderately high bilirubin levels without a recognizable bilirubin encephalopathy in neonatal periods, had late and mild abnormal neurological deficits such as delay motor development, cognitive abnormality, autism, attention deficit hyperactivity disorder (ADHD). Although these deficits have modest intensity, more research is needed for confirmation of the exact correlation between these late sequelae of neonatal hyperbilirubinemia** (**24**).**

   **Clinical manifestations**

Transient bilirubin encephalopathy

In some newborns with high levels of bilirubin, early bilirubin toxicity is reversible and transient. There are several case reports in neonates with moderate to severe hyperbilirubinemia of resolution of acute bilirubin encephalopathy without progression to kernicterus (3). In these lucky patients, despite serious risk factors to extreme hyperbilirubinemia, timely and appropriate treatment of jaundice by intensive phototherapy and exchange transfusion, stop the process of bilirubin toxicity in the brain. The reversible and short duration of symptoms such as lethargy and abnormal auditory brainstem responses, after the exchange transfusion, are the clinical manifestations of this condition **(**19**,**^25^,^4^**).**

**Bilirubin induced neurological dysfunction (BIND)**

Bilirubin toxicity in a less severe intensity than kernicterus with a minor neurological dysfunction, defined as BIND. Subtle disorders of vision (vasomotor dysfunctions), hearing, neuromotor function, speech, cognition and language, muscle tone abnormalities are the characteristics of this complication. Hyper excitable neonatal reflexes and a variety of neurobehavioral manifestations are the other symptoms of this condition **(**26**).**

In this situation, neonates exposed to bilirubin levels of lesser severity than in classic kernicterus.

**Acute bilirubin encephalopathy (ABE)**

The acute symptoms of disease divided into 3 phases which are listed below:

**Early phase**: In the first 3-5 days of the disease, the nonspecific symptoms of slight lethargy, poor feeding, poor sucking, slight hypotonia and hyperreflexia, slightly high pitch cry are seen. These findings are similar to some other common problems in neonatal periods such as sepsis, hypoglycemia, hypothermia, and IVH.

**Figure-2:**
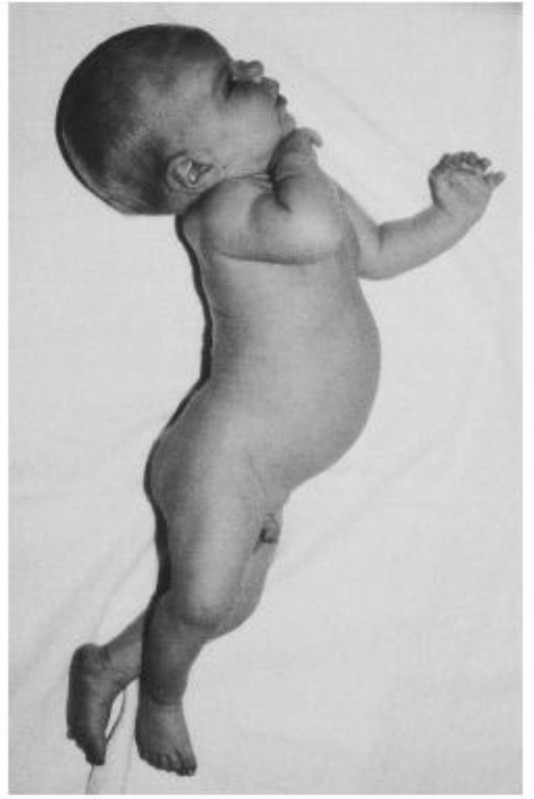
Back arching and opisthotonos in a 1-month-old neonate with kernicterus. The kernicterus was secondary to Crigler-Najjar syndrome

**Intermediate phase**: At the end of 1st week of life acute bilirubin encephalopathy presented with moderate stupor, irritability, fever, hypo and hypertonia as an alternative symptom, back arching and hyperextension of extensor muscles (opisthotonos, retrocollis) and high-pitched cry (Figure -2**).**

**Late (advanced) phase**: In this phase, deep stupor or coma, high pitch crying, pronounced retrocollis-opisthotonos, and no feeding is observed.

Hearing and visual abnormality, athetosis, and seizure (in some cases) were seen after the first week of life, also the hypotonia can be the predominant symptom of the disease in this phase** (**3**).** 

Seizure is seen in <50% of patients. The persistent seizure is not a common finding of neonates with kernicterus and in most of the patients, seizure resolved several weeks after the acute phase of the disease (19).

Definitive neurologic signs of acute bilirubin encephalopathy are seen in 55-60%of the patients. Other cases, can be having no definitive symptoms or have equivocal neurologic signs.

As regards to the immaturity of the nervous system in preterm babies, the symptoms of bilirubin encephalopathy of the premature neonates are subtle and nonspecific. Behavior change, cardiorespiratory instability, central processing disorders, extrapyramidal tone abnormality are the finding of bilirubin toxicity in premature neonates.** (**27**)**

Mortality related to severe bilirubin toxicity is reported in 23% of neonatal death and probably much more in preterm babies**. (**28**)**

(Courtesy Dr. M. Jeffrey Maisels.)

**Chronic bilirubin encephalopathy**

In the first year of life hypotonia, hyperreflexia, delayed motor skill, persistent tonic neck reflex, and retardation of neurodevelopmental milestones are present. Extrapyramidal movements well developed after several years. During the late infancy and childhood, a tetrad of symptoms including visual (upward gaze palsy), auditory (sensory neural hearing loss), dental dysplasia abnormalities, and extrapyramidal disturbances (choreoathetosis cerebral palsy) are presented in the patients**. (**29**) **Intellect is relatively spared.

 **Auditory dysfunction**: Auditory brainstem center is the first and the most affected structure in the patient by BIE. After that, the auditory nerve is involved but, the cochlea and hair cells spared.** (**30**)** Then the hearing loss due to bilirubin encephalopathy is central (brain stem) and in a lesser extent has a peripheral origin**.** Sensorineural hearing loss may be the only symptom of bilirubin encephalopathy. In most cases, the auditory disturbance is a high-frequency loss, and usually is bilateral. Treatment of hyperbilirubinemia led to a considerable decrease in the incidence of hearing loss.

Auditory neuropathy or auditory dyssynchrony is one of the other hearing problems with bilirubin toxicity which differs from typical hearing loss. In this situation, there is little or no hearing loss, but abnormal processing of sound is seen. Sound localization and speech discrimination is the product of this disorder. 

**Figure -3):**
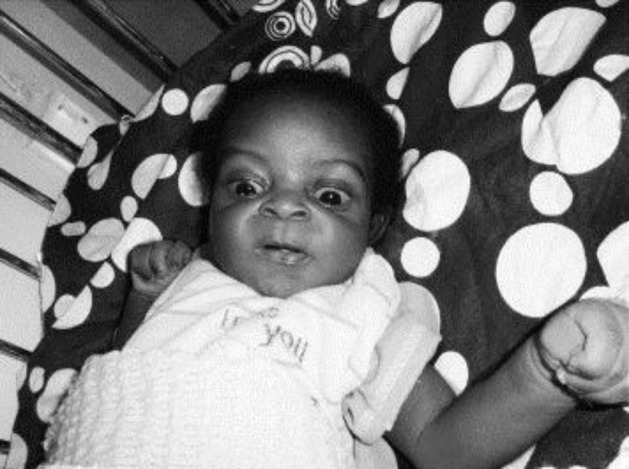
kernicterus face, Sixth Gree

 Horizontal gaze dysfunction. Paralysis of upward gaze and a blank stare or “scared appearance” caused by the combination of upward gaze paresis and facial dystonia are the visual effects of bilirubin encephalopathy. Due to this involvement and based on the appearance of patients, kernicterus face is defined which includes eyelid retraction and the setting-sun sign (i.e., paresis of upward gaze) which together comprise the Collier sign, and facial dystonia. These findings make the infant appear scared, or anxious and stunned, some infants may have wondering or disconjugate eyes. Sometimes both horizontal and vertical eye movements are affected. This kernicterus appearance and faces (Figure -3) persist for at least two to three weeks after acute bilirubin encephalopathy **(**31**).** 

Enamel dysplasia: Greenstick teeth and dental enamel dysplasia are the other complications of bilirubin encephalopathy (Figure- 4). This complication is an extremely uncommon abnormality that can affect both primary and permanent teeth. With excessive hyperbilirubinemia, reversible staining of all tissues of the body occurs except for the teeth. Due to loss of metabolic activity after maturation, the bile-pigments are permanently trapped. The pigmentation of the teeth may vary from yellow to deep shades of green. (32**)**

**Figure-4:**
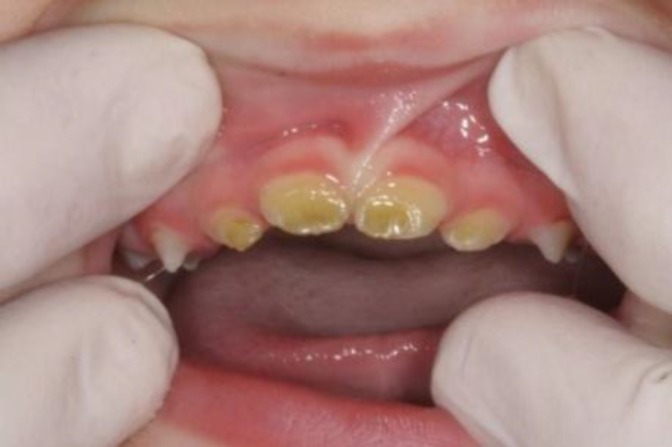
Enamel dysplasia as a complication of BIE, Barberio GS

**Extrapyramidal syndrome**: The involvement of basal ganglia and Globus pallidus and subthalamic nuclei present with extrapyramidal dysfunctions. Dystonia and choreoathetosis are the main symptoms of this disorder.** (**33**)** Nearly all patients with kernicterus present variable degrees of this movement disorder. In the most severe cases, choreoathetosis present with Upper and lower extremities involvement, but upper extremities affected more severe. Limb movement exhibit as chorea (rapid and jerky movements), tremor or ballismus (wide amplitude flailing). Dystonic posture is common.

 Bulbar and cognitive dysfunctions, delayed neuromotor/psychomotor development are the other rare complications **(**34**).** 

**Extra neural bilirubin toxicity**: Deposition of indirect bilirubin in other organs than the brain such as pancreatic cells, renal tubular cells, and intestinal mucosa is seen in the autopsy of the patients. Long term consequences of bilirubin toxicity in these organs are not specified.

**Diagnosis:**

Regard to the inability to t observation of deposited bilirubin in the brain nuclei, the diagnosis of bilirubin encephalopathy can be done by the clinical characteristics of the patients, abnormal imaging on the brain MRI, and abnormal findings on the auditory brainstem responses. Some clinical tools using abnormal physical findings and grads the severity of the disease. BIND (Bilirubin induced neurological dysfunction**)** score is designed for this purpose. BIND score is an objective tool for assessment the level of neurological involvement of the patient. Mental status, muscle tone and cry characteristics are the three parameters for the categorize the patients at the three levels of involvement: subtle (1-3 score), moderate (4-6 score) and advance (7-9 score). The score of 0 signify the normal newborn and low score indicate of normal neurologic outcome. But patients with high score died or have residual neurologic dysfunctions**. (**15**)** In the modified BIND score with 12-point score, the eye characteristics of the patient: divergent gaze, paralysis of upward gaze, anxious appearance and nystagmus were added in the scoring system. This modification helps to differentiate the bilirubin encephalopathy from the other etiologies with similar neurologic findings such as tetanus.

Positive and negative predictive value of this scoring system in the diagnosis and grading of bilirubin encephalopathy is 88.9% and 98.2% respectively.** (**35**)**

**Table1:** Bind Score

**Clinical Parameter**	**BIND Score**
**Mental status**	
Normal	0
Sleep but arousable, decreased feeding	1
Lethargy, poor suck and/or irritable/jittery with strong suck	2
Semi-coma, unable to feed, seizures, coma	3
**Muscle tone**	
Normal	0
Persistent mild to moderate hypotonia	1
Hypertonia alternating with hypotonia, beginning arching of neck and trunk on stimulation	2
Persistent retrocollis and opisthotonos—bicycling or twitching of hands and feet	3
**Cry pattern**	
Normal	0
High pitched when aroused	1
Shrill, difficult to console	2
Inconsolable crying or cry weak or absent	3
Eye movements	
Normal	0
Divergent gaze	1
Paralysis of upward gaze	2
Anxious appearance and nystagmus	3
**TOTAL **12	

**Brainstem auditory evoked response (BAER):** Regard to the hearing impairment is the most common complications of bilirubin encephalopathy, ABR known as a noninvasive and available modality for the early diagnosis of acute bilirubin encephalopathy with a 100% sensitivity and 99.4%specificity.ABR can be used as a predictive tool for bilirubin encephalopathy** (**36**).** Bilirubin induced auditory neuropathy occurs in spite of normal cochlear function as detected in otoacoustic emission (OAE). Then in all neonates with extreme hyperbilirubinemia BAER is necessary.

**Figure -5:**
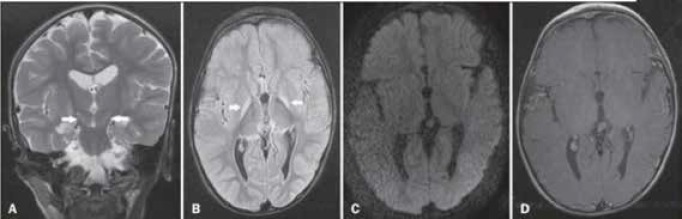
A: Coronal T2-weighted MRI sequence showing a bilateral, symmetrical hyperintense signal in the subthalamic nuclei (arrows), without a mass effect. B: Axial FLAIR MRI sequence showing a bilateral, symmetrical hyperintense signal in the globus pallidus (arrows). C: Axial diffusion-weighted MRI sequence showing no diffusion restriction. D: Axial T1-weighted MRI sequence showing no evidence of gadolinium enhancement

**Neuroimaging finding**: MRI is the most valuable radiologic modality for diagnosing the acute and chronic neurologic sequels of bilirubin encephalopathy.  The acute lesions of this disorder are transient and disappeared later. Involvement of Globus pallidus in 90% of patients, after that sub thalamic area in 40% of cases observed. In the first several weeks of disease, the best imaging of MRI is recognized in T1 –weighted images and in chronic bilirubin encephalopathy, T2 –weighted images are the best one (Figure -5). Generalized brain edema in acute phase and bilateral, symmetrical hyper intensity in T2, flair in Globus pallidus and subthalami nuclei in chronic phase are characterized** (**37**).**

Management

In the acute phase of bilirubin encephalopathy, early intervention of severe hyperbilirubinemia with phototherapy and exchange transfusion can be prevented the progression of bilirubin toxicity. Until now there is no definitive information about the exact duration time of severe hyperbilirubinemia and the initiation of bilirubin encephalopathy. What’s certain, in cases with extreme hyperbilirubinemia especially in sick and preterm babies and in neonates with underlying hemolytic disease, delay initiation of treatment for more than a few hours increase the probability of BIE. Although the treatment of hyperbilirubinemia is specified, unfortunately, there is no certain cure for the consequences of bilirubin encephalopathy and rehabilitation is the only therapeutic strategy. Then the best strategy is the prevention of severe hyperbilirubinemia with the timely diagnosis and treatment of jaundice. (38) Children with auditory neuropathy and hearing loss appear to respond favorably to cochlear implantation.

**Novel treatments for prevention of BIE**

There are many ongoing studies for the evaluation of the new therapeutic strategies to reduce the long-term neurologic consequences of BIE. New treatment modalities such as Minocycline as an anti-inflammatory and antimicrobial drug, therapeutic hypothermia for making mitochondria more efficient as a main biological factor for bilirubin induced brain cell injury, caffeine in bilirubin toxicity of preterm neonates, anti-apoptotic agents, antioxidant therapies (free radical scavengers) 

or stem cell treatment as a final therapeutic option are introduced. Future high-quality studies help to select the best strategy for the prevention of neurological complications of kernicterus **(**39).

 **Prevention**

 The most effective way to eliminate the BIE is the prevention of excessive hyperbilirubinemia in neonatal periods. In fact, the prevention of bilirubin toxicity can be started from the perinatal periods by the screening of high-risk families with Rh-negative mothers and Rh-positive fathers, administration of RhoGam injection for the prevention of maternal hypersensitivity and resulted in fetal hydrops fetalis and neonatal severe hyperbilirubinemia. After birth, at the nursery before the discharge from the hospital, detection of high-risk neonates for severe hyperbilirubinemia and planning for close observation and follow up after discharge from the nursery can be successful for the timely diagnosis of hyperbilirubinemia in the early phase of the disease. In this situation, timely treatment by phototherapy or exchange transfusion can prevent the BIE even in neonates with high predisposing factors for extreme hyperbilirubinemia.

**In Conclusion, **Despite the identified treatment of neonatal hyperbilirubinemia, BIE continues to occur and early detection of high-risk neonates for severe jaundice is the best modality for the prevention of this disorder. BIE has a wide spectrum of symptoms consisted of mild neurologic disorder to classic kernicterus. It’s very important to mention that just measuring the total bilirubin level is not the most sensitive marker for the prediction of bilirubin toxicity. Although the hearing impairment is the most common complications of BIE ,the neurological dysfunctions of the patients has a wide spectrum of involvements and all patients do not have the all tetrad of symptoms including visual (upward gaze palsy), auditory (sensory neural hearing loss), dental dysplasia abnormalities, and extrapyramidal disturbances (choreoathetosis cerebral palsy). Regard to the normal intelligent quotient (IQ) in most of the patients with kernicterus, auditory and motor dysfunction of the patient must not mistake with severe cognitive impairment
